# Degassing a Decellularized Scaffold Enhances Wound Healing and Reduces Fibrosis during Tracheal Defect Reconstruction: A Preliminary Animal Study

**DOI:** 10.3390/jfb14030147

**Published:** 2023-03-05

**Authors:** Nguyen-Kieu Viet-Nhi, Yen-Chun Chen, Luong Huu Dang, How Tseng, Shih-Han Hung

**Affiliations:** 1 International Master/Ph.D. Program in Medicine, College of Medicine, Taipei Medical University, Taipei 110, Taiwan; 2 Department of Otolaryngology, Taipei Medical University Hospital, Taipei 110, Taiwan; 3 Graduate Institute of Medical Sciences, College of Medicine, Taipei Medical University, Taipei 110, Taiwan; 4 Department of Otolaryngology, Faculty of Medicine, University of Medicine and Pharmacy at Ho Chi Minh City, Ho Chi Minh City 70000, Vietnam; 5 Department of Biochemistry and Molecular Cell Biology, School of Medicine, College of Medicine, Taipei Medical University, Taipei 110, Taiwan; 6 Department of Otolaryngology, School of Medicine, College of Medicine, Taipei Medical University, Taipei 110, Taiwan; 7 Department of Otolaryngology, Wan Fang Hospital, Taipei Medical University, Taipei 116, Taiwan

**Keywords:** tissue engineering, small intestinal submucosa, degas, tracheal patch model

## Abstract

Few efforts have been made regarding the optimization of porcine small intestinal submucosa (SIS) to improve its biocompatibility. This study aims to evaluate the effect of SIS degassing on the promotion of cell attachment and wound healing. The degassed SIS was evaluated in vitro and in vivo, compared with the nondegassed SIS control. In the cell sheet reattachment model, the reattached cell sheet coverage was significantly higher in the degassed SIS group than in the nondegassed group. Cell sheet viability was also significantly higher in the SIS group than in the control group. In vivo studies showed that the tracheal defect repaired by the degassed SIS patch showed enhanced healing and reductions in fibrosis and luminal stenosis compared to the nondegassed SIS control group, with the thickness of the transplanted grafts in the degassed SIS group significantly lower than those in the control group (346.82 ± 28.02 µm vs. 771.29 ± 20.41 µm, *p* < 0.05). Degassing the SIS mesh significantly promoted cell sheet attachment and wound healing by reducing luminal fibrosis and stenosis compared to the nondegassed control SIS. The results suggest that the degassing processing might be a simple and effective way to improve the biocompatibility of SIS.

## 1. Introduction

The use of decellularized tissue during surgical procedures in humans for repair and reconstruction has been made possible by using an SIS mesh derived from the porcine small intestinal submucosa (SIS) [1,2,3,4]. SIS mesh is composed primarily of extracellular matrix (ECM) without cellular contents and thus can be widely used for soft tissue repair in many surgeries [5]. However, there have been few explorations on the optimal use of SIS mesh to increase its biocompatibility.

Ever since 1998, when SIS was cleared by the FDA for its first clinical applications in wound repair [6], ECM-based porcine SIS has exhibited good biocompatibility and low immunogenicity when reconstructing various types of tissues, including those involving urological diseases such as hypospadias [2] and urinary bladder reconstruction after cystectomy [7]; gynecological illnesses such as cervicovaginal reconstruction [4] and pelvic organ prolapse [8]; and chronic poor healing wounds such as diabetic foot ulcers [9] and stage III or IV pressure ulcers [10]. Moreover, SIS has been used for focal tissue repair in the eardrum [1] and heart valves [11] and for bone augmentation in animal studies [12]. However, the outcomes remain uncertain in several fields in which SIS is used [8,11]. For instance, one retrospective study using SIS in the repair of pelvic organ prolapse revealed the relatively high complication rates (56%) after the operations, leading to the suggestion of little benefit of SIS graft in prolapse surgery [8]. Another study in the pediatric congenital aortic valve repair found a shorter time interval to reintervention and significantly higher odds ratio of the occurrence of moderate aortic regurgitation or stenosis when using SIS compared to the use of autologous pericardium [11]. While SIS holds the potential to promote constructive remodeling at site-appropriate functional tissue [13], whether this material needs to be pretreated or surface-modified before site-specific implantation to increase clinical efficacy warrants more investigation.

In tissue engineering, a degassing process is usually adopted to remove cellular contents from an ECM-based scaffold or the air bubbles inside a porous material. Two studies reported that decellularization of porcine tracheal scaffolds using a combined vacuum–enzyme/detergent protocol significantly decreased the fabrication time to 9 days compared to 3–8 weeks without vacuum assistance [14,15]. Another study pointed out the benefit of applying vacuum pressure to increase the penetration of collagen into a poly-L-lactic acid (PLA) scaffold, in which the resultant PLA–collagen composite scaffold showed improved water adsorption and degradation [16].

Currently, degassing processes, or vacuum-assisted methods, are often used to shorten the preparation time and eliminate residual cellular contents in tissue engineering [17,18,19]. Luo et al. showed that the efficiency of decellularization in heart valves treated under vacuum was enhanced, and the elasticity and tensile strength after the decellularization process remained uncompromised [17]. Furthermore, another study demonstrated that the preparation of decellularized tracheal scaffolds with vacuum assistance and optimal DNase I concentration could achieve good effects of decellularization within only two days [19]. In comparison to other previously reported methods of surface modification in SIS such as functional group bonding, protein adsorption, mineral coating, or topography and formatting modifications [20], the relatively simple and practicable degassing process might hold potential in increasing the efficiency of SIS in tracheal luminal wound healing. The study by Negishi et al. [16] presented a method to overcome the difficulty of incorporating the heat-sensitive natural polymer collagen into a PLA scaffold by using a vacuum pressure impregnation method. This encouraged us to consider whether the process of degassing would enhance cellular adhesion and proliferation and therefore enhance the biocompatibility and clinical effectiveness of SIS.

This study aims to evaluate the efficacy of degassed SIS on the promotion of cell attachment and wound healing and the reduction of fibrosis in an in vitro cell sheet reattachment model and an in vivo tracheal patch defect repair model.

## 2. Materials and Methods

**Figure 1:**
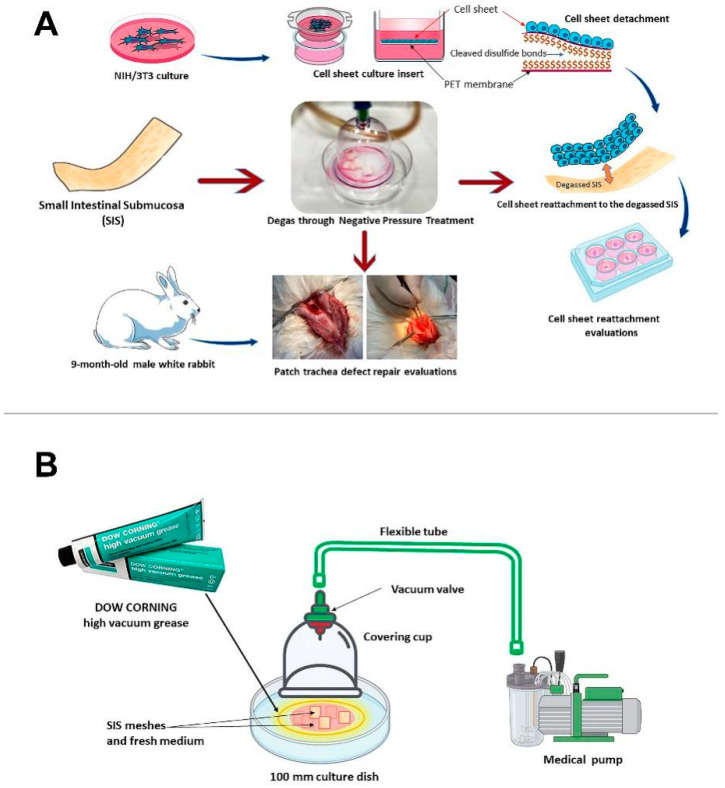
Flow diagram of the research design (**A**). Schematic diagram of the vacuum system (**B**).

The study flow diagram is shown in Figure 1A. In brief, degassed SIS was subjected to an in vitro cell attachment ability test with an NIH-3T3 cell sheet. Following the in vitro evaluation, degassed SIS was evaluated for its ability to facilitate wound healing and reduce fibrosis in a rabbit trachea patch repair model.

### 2.1. SIS Mesh Preparation and Degassing

#### 2.1.1. SIS Mesh Preparation

DynaMatrix Plus produced by Cook Biotech Incorporated (1425 Innovation Place, West Lafayette, IN 47906, USA) was used in this study. DynaMatrix Plus is specifically designed to serve as a bioactive soft tissue regeneration product for augmentation procedures. The qualified pig’s small intestinal submucosa (SIS) was harvested and fabricated into an extracellular membrane. The natural composition of matrix molecules such as collagen (types I, III, and IV), glycosaminoglycans (hyaluronic acid, chondroitin sulfates A and B, heparin, and heparan sulfate), proteoglycans, growth factors (FGF-2, TGF-β), and fibronectin were retained in the SIS derivation process. After purchase, the sterilized SIS scaffolds were cut into smaller pieces (10 mm × 10 mm) and divided into two groups and treated with or without degassing.

#### 2.1.2. Degassing of the SIS Scaffold

A custom-designed vacuum system with a covering cup, medical pump, flexible tubes, and cell culture dishes was used in the degassing process in this study (Figure 1B). First, all devices were sterilized with 75% alcohol and UV irradiation for 15 min. Six pieces of SIS material were placed into a 100 mm culture dish (diameter 100 mm, surface area 56.7 cm^2^). Then, 2 mL of fresh DMEM (Gibco, Life Technologies Corporation, 3175 Staley Rd., Grand Island, NY 14072, USA) containing 10% fetal bovine serum (EDM Millipore Corp., 290 Concord Rd, Billerica, MA 01821-3405, USA) and 1% penicillin-streptomycin (Gibco, USA) were added to the 100 mm culture dishes. Next, the cup (diameter 45 mm, surface area 15.9 cm^2^) was placed on the 100 mm culture dish, and the contour of the cup was pressed down slightly to the surface of the 100 mm culture dish. A specific DOW CORNING high vacuum grease (DOW CORNING Corporation, Midland, MI 48686-0994, USA) was used between the contour of the cup and the surface of the 100 mm culture dish. A sterilized flexible tube was used to connect the valve of cup to a standard medical portable suction machine (SPARMAX, Taipei 110, Taiwan) located outside of the hood. The input operating vacuum was set to 650 mm Hg, and the output airflow was set to 20 LPM (liters per min) over 20 min.

### 2.2. Cell Sheet Fabrication

#### 2.2.1. Preparation of the Cell Culture Inserts

According to our previous publication, we used porous polyethylene terephthalate (PET) membranes for chemical surface modification [21,22]. Solutions one and two were prepared by dissolving 0.125 M hyaluronic acid (Kewpie, Japan) in 0.25 M boric acid buffer and EDC/NHS/cystamine (ACROS Organics, Belgium) in 0.25 M boric acid buffer. The final solution (three) was prepared by pouring solution one into solution two for two hours of reaction. Then, a 6-well culture insert with a porous PET membrane (pore size: 430 nm, pore density: 5.63 × 10^6^/cm^2^, thickness: 12.5 μm) (ANT Technology, Taiwan) was preactivated with low-pressure plasma (PDC-002-HP, Harrick Plasma, USA) at 500 mTorr for 45 min under a carbon dioxide atmosphere. After immersing the PET culture insert in 0.25 M EDC/NHS in 0.25 M boric acid buffer at pH 6.0 and 4 °C for 2 h, the insert was mixed with an equal volume of solution. Continuous shaking was then performed for 4 h. The culture inserts containing HA-modified porous HA-PET with a disulfide bond were gently washed with water and kept dry overnight. Finally, the inserts were sterilized with ethylene oxide gas for future use in culture.

#### 2.2.2. Cell Sheet Culture

In our previous attempts (unpublished data), we found that nasal epithelial primary cell cultures differ significantly from other cell lines in their ability to attach. Therefore, in our current study, instead of a direct cell seeding model, a cell sheet detachment and reattachment model was used to reveal the effectiveness of degassing the SIS surface as in living healing conditions; the tissue and the SIS interact through surface-to-surface contact. The surface reattachment ability might more accurately reveal the effectiveness of degassing the SIS in promoting tissue healing instead of seeding individual cells on the SIS surface. The NIH/3T3 cell line was chosen based on its rapidly growing property and is relatively stable in creating a condition similar to the cell sheet for reattachment comparison purposes.

The NIH/3T3 (mouse) fibroblast cells were purchased from the Bioresource Collection and Research Center, Hsinchu, Taiwan (BCRC no. 60008) and used in this study. A monolayer of the cells was cultured in a 60 mm culture dish (AlphaPlus, Taiwan) in fresh 3T3 medium containing DMEM (Gibco, USA), 10% fetal bovine serum (EDM Millipore Corp., MA 01821-3405, USA), and 1% penicillin-streptomycin (Gibco, USA). The medium was replaced every 3 days, and the cells were maintained in an incubator at 37 °C with 5% CO_2_.

When the density reached approximately 80% confluence, the 3T3 cells were detached with 0.25% trypsin–EDTA (Gibco, USA). Then, the obtained cells were seeded on the cell culture inserts (surface area 3.5 cm^2^/insert) at a density of 5 × 10^5^ cells/insert in fresh 3T3 medium.

After 10 days of culture, the 3T3 cell sheets were harvested from the inserts by adding 5 mL of reducing agent solution, a mixture of 0.279 g of L-cysteine in 0.5 mL of 1 N NaOH and 29.5 mL of PBS.

#### 2.2.3. NIH/3T3 Cell Sheet Reattachment to the Scaffold

The degassed scaffolds were placed into a new 6-well culture plate. Then, medical tweezers were used to move the harvested cell sheets to the 6-well culture plate. Initially, a few volumes of medium were added to the 6-well culture plate. The 3T3 cell sheets reattached to scaffolds were incubated in the incubator (+37 °C, 5% CO_2_) for one hour, and then more medium was gently added for two weeks of further culture.

The nondegassed scaffolds, as the control group, underwent a similar procedure.

### 2.3. In Vitro Evaluation of Degassed SIS Mesh Cell Sheet Attachment

#### 2.3.1. Reattached Cell Sheet Surface Analysis

After the incubation period, the old medium was removed, and the reattached 3T3 cell sheets were washed twice from the SIS surface. The cell sheet scaffolds underwent the shaking test and the rinsing test. First, the cell sheet scaffolds were shaken by a shaking machine for 10 min at 100 rpm. Then, they were held by medical tweezers, inclined 45 degrees to the surface of the culture plate, and rinsed under PBS solution flow five times. After rinsing, the cell sheet scaffolds were placed into another 6-well plate culture.

#### 2.3.2. Reattached Cell Sheet MTT Assay

First, 200 μL of MTT reagent (MedChemExpress Co., Ltd., 1 Deer Park Dr, Suite Q, Monmouth Junction, NJ 08852, USA) (final concentration of 0.5 mg/mL) was added to each well containing the reattached SIS cell sheet material, and the plate was placed in an incubator (+37 °C, 5% CO_2_). After four hours, the MTT solution was removed. Purple crystals were observed on the surface of the SIS material. A standard light was used to take photos of the attached SIS cell sheet. Then, the attachment areas were analyzed by the image processing software ImageJ (version 1.43u) developed by the National Institutes of Health (USA). The color threshold was set to accurately capture the purple area of the MTT-stained attached cell sheet without picking up any signals in the control group (group without the cell sheet).

Next, 200 μL of DMSO solution was added to each well. After that, the plates were incubated in the incubator for 10 min (+37 °C, 5% CO_2_). It was verified that the purple formazan crystals had been completely solubilized, and the absorbance of each sample was measured spectrophotometrically at 570 nm by a Tecan Spark™ 10 M multimode microplate ELISA reader.

#### 2.3.3. H&E Staining

For histological analysis, the reattached cell sheet samples were fixed in a 10% neutral buffered formalin solution in PBS (pH 7.4) at room temperature for 20 min, washed with PBS 3 times, dehydrated in graded alcohol, embedded in paraffin (Merck, Darmstadt, Germany), and sectioned at 5 µm. Adjacent sections were stained with hematoxylin and eosin (H&E) (Sigma, USA) and observed under a microscope (OLYMPUS BX53, Japan).

### 2.4. In Vivo Evaluation of the Degassed SIS Mesh in a Trachea Patch Repair Model

#### 2.4.1. Ethics Statement and Animal Use

The following animal handling procedure was reviewed and approved by the Institutional Animal Care and Use Committee of Taipei Medical University (approval no. LAC-2020-0173). Ten 9-month-old male New Zealand white rabbits (supplied by BioLASCO Taiwan Co., Ltd., Taipei City, Taiwan) with body weights between 3 kg and 3.5 kg were included in this study. The rabbits were housed individually under standard conditions (22–24 °C, exposed to cycles consisting of 12 h of light and 12 h of dark, and allowed free access to food and water). Six hours before anesthesia, the rabbits were provided a light meal, but water was provided ad libitum. Prior to surgery, the rabbits were weighed and then intramuscularly injected with 0.1 mL/kg Zoletil, which contains 50 mg/mL tiletamine and 50 mg/mL zolazepam (Zoletil^®^ 100; Virbac, Carros, France) and 0.4 mL/kg xylazine (^®^Rompun 20 mg/mL; Bayer HealthCare, LLC, Animal Health Division, Shawnee Mission, KS 66201, USA) to induce short-term anesthesia. Rabbits were intubated and constantly monitored during the course of anesthesia for level of consciousness and any signs of discomfort. Removal of the intubation tube was attempted when the animal regained consciousness and began rejecting the tracheal tube. All reasonable actions were taken to minimize suffering throughout the operation. Rabbits were euthanized at either the end of the experiments or when a humane endpoint was reached, whichever came first. Humane endpoints for all experiments were defined as 20% acute weight loss or clinical signs consistent with severe dyspnea, altered mentation, or anorexia.

#### 2.4.2. Patch Model

After assessing the capacity of the degassed SIS mesh to promote cell adhesion and proliferation, we then applied the degassed SIS mesh to reconstruct tracheal defects. The patch defect model was constructed in 10 rabbits that were able to adequately before the investigation. After the trachea was accessible, the ventral portion, which had a semicylindrical shape and measured approximately 0.7 cm × 0.7 cm, was excised. On five rabbits, a degassed SIS mesh patch of the same size as the wound was sutured in place using a nonabsorbable surgical suture (Prolene^®^ 6-0; ETHICON, LLC., San Lorenzo, Puerto Rico 00754-0982, USA) to reconstruct the defect. The muscle was closed with two sutures (Vicryl^®^ 4-0; ETHICON, USA), followed by S.C. skin closure (Nylon^®^ 4-0; ETHICON, USA) Five other rabbits underwent the same procedure, but the original SIS mesh was sutured in place of the defect as the control group.

#### 2.4.3. Histological Analysis

After administering Zoletil (Zoletil^®^ 100; Virbac, Carros, France) intramuscularly to induce general anesthesia, euthanasia was carried out using carbon dioxide gas. After that, the transplanted section was promptly removed together with the host tracheal structures for gross and histological analyses. The explanted specimens were dissected to remove all surrounding tissues to expose the cartilage tube structure. Subsequently, the samples were fixed for 24 h in a 10% neutral buffered formalin solution in PBS (pH 7.4) at room temperature, rinsed with distilled water, dehydrated in graded alcohol, and embedded in paraffin. Paraffin blocks were cut into 4 μm sections and stained with a hematoxylin-eosin staining solution (Sigma-Aldrich, MI, USA). Using a light microscope (Axioskop; Carl Zeiss, Oberkochen, Germany) at 100× magnification, microscopic quantification was performed by one researcher blinded to the experimental groups. The thickness of the tracheal wall at the implanted defect was measured.

### 2.5. Statistical Analysis

ImageJ software (National Institute of Health, New York, NY, USA) was used to measure the thickness of each implanted graft. Statistical analyses were carried out using Prism version 5 (GraphPad Software, CA, USA). Differences in the thicknesses of the mucosal layers of the grafted patches with and without cell sheet application were assessed by unpaired Student’s *t* test. A *p* value < 0.05 was considered significant and noted as *p* < 0.05 (*), *p* < 0.001 (***), and *p* < 0.0001 (****).

## 3. Results

### 3.1. In Vitro Evaluation of the Ability of the Degassed SIS Mesh Cell Sheet to Attach

**Figure 2:**
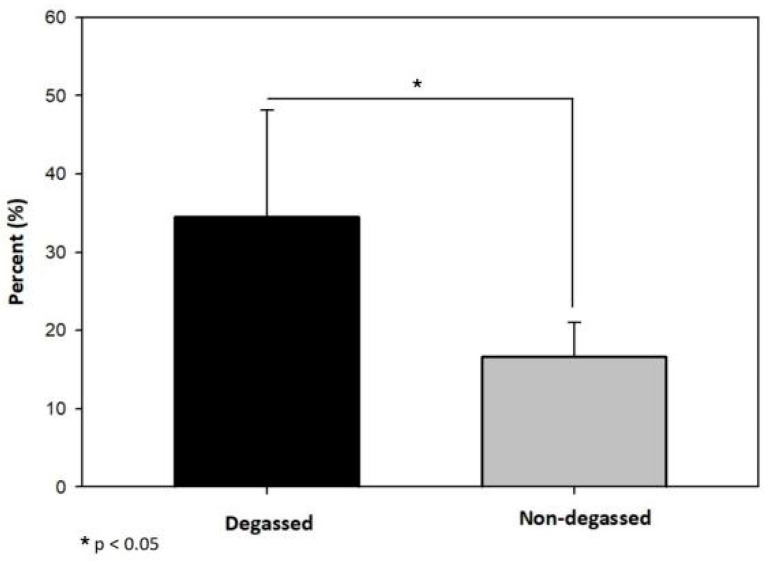
Reattachment surface area analysis of the degassed SIS mesh. The area of reattached cell sheets in the degassed group was 34.57 ± 11.8%, which was significantly higher than that in the nontreated group (16.72 ± 3.8%, * *p* < 0.05).

Upon analysis of the MTT-stained reattached cell sheet images, the percentage of the area of the reattached cell sheets in the degassed group was 34.57 ± 11.8%, which was significantly higher than the 16.72 ± 3.8% in the nontreated group (*p* < 0.05) (Figure 2).

**Figure 3:**
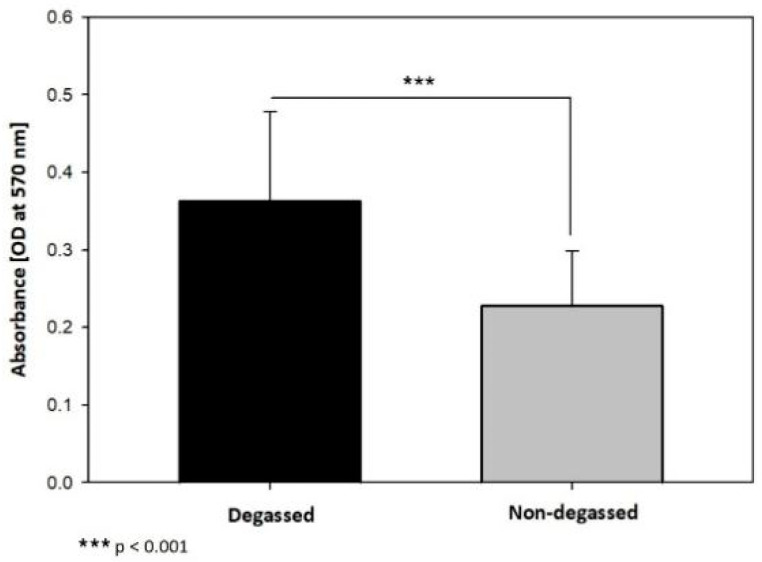
Viability of the reattached cells in the degassed SIS mesh. The optical density (OD) measured by the ELISA reader in the degassed group was 0.363 ± 0.116, which was significantly higher than that in the nontreated group (0.228 ± 0.072, *** *p* < 0.001).

The degassed group had more live reattached cell sheets than the untreated group. The absorbance values of the samples were calculated and analyzed using the independent t test. The optical density (OD) detected by the ELISA reader in the degassed group was 0.363 ± 0.116, which was significantly higher than the 0.228 ± 0.072 of the nontreated group (*** *p* < 0.001) (Figure 3).

**Figure 4:**
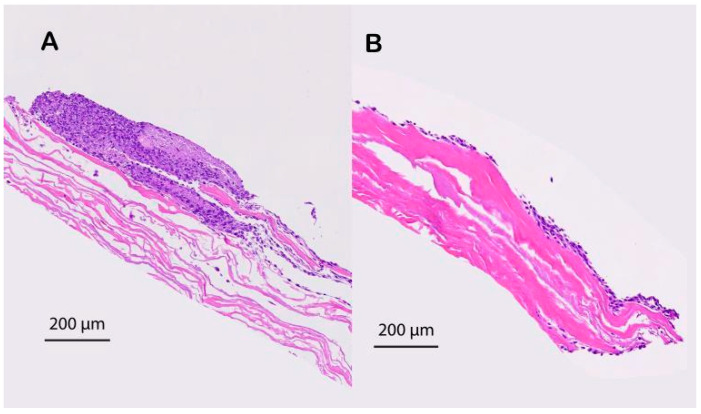
Histological comparison of the cell sheet reattached to the degassed SIS mesh. The cell sheet that reattached to the degassed SIS adhered to the surface very well. (**A**) No voids were observed between the two layers compare to the nondegassed control (**B**).

The HE-stained specimens showed that the fabricated scaffolds consisting of cell sheets that had reattached during vacuum treatment could adhere to the surface of the SIS since no voids were observed between the two layers (Figure 4).

### 3.2. In Vivo Evaluation of the Degassed SIS Mesh in a Trachea Patch Repair Model

**Figure 5:**
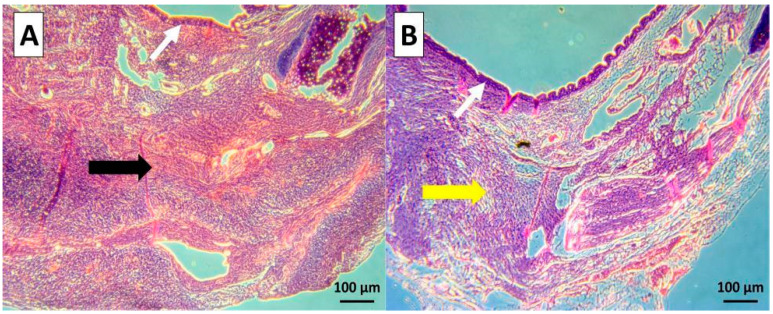
In vivo evaluation of the degassed SIS mesh in a trachea patch repair model: (**A**) H&E staining: the nondegassed control group showed dense fibrosis with high neovascularization (black arrow). (**B**) H&E staining: the DSA-treated experimental group showed significant epithelial regeneration (white arrow) and less fibrosis formation around the transplanted sheet (yellow arrow).

**Figure 6:**
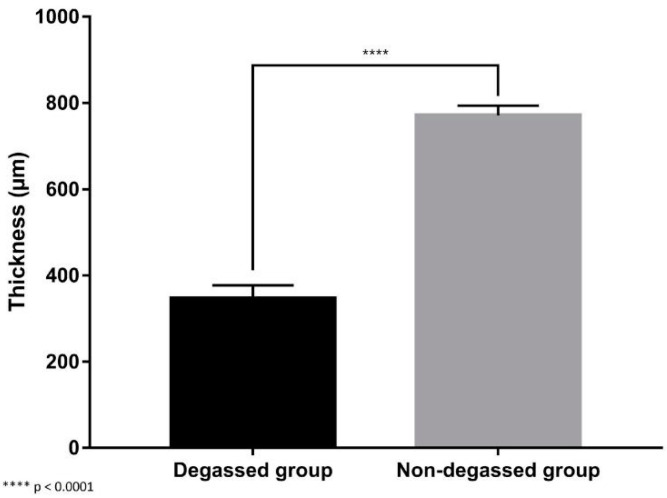
Image analysis of the measured thickness of the mucosal layer of each implanted graft. There was a significant reduction in the thickness of the transplanted degassed SIS mesh graft compared with and the nondegassed SIS mesh (346.82 ± 28.02 µm vs. 771.29 ± 20.41 µm, respectively; **** *p* < 0.0001).

No animals died during the surgical procedure. The surviving rabbits did not exhibit any clinical symptoms of respiratory difficulty, and euthanasia was performed in time. As the implanted site is deep in the tracheal lumen, we found it difficult to observe the progress of the thickness of the implanted scaffold continuously. Instead, the thickness of the implanted graft of the two groups at the time of two months postoperation was compared. At two months postoperation, histological assessment showed that the areas that were transplanted with the graft in both experimental groups had an intact epithelium. However, the tracheal defect repaired by the degassed SIS patch showed enhanced healing and reductions in fibrosis and luminal stenosis compared to the nondegassed control group. In the control group, we observed dense fibrosis, high neovascularization in the subepithelial layer, and a large amount of fibrosis formation at the contact site where the SIS mesh had been implanted (Figure 5). Conversely, the degassed SIS patch showed better incorporation into the transplanted site, with less lymphocyte infiltration and less fibrosis formation. As a result, there was a significant reduction in the thickness of the degassed SIS transplanted graft compared with the nondegassed SIS graft (346.82 ± 28.02 µm vs. 771.29 ± 20.41 µm, respectively; *p* < 0.05) (Figure 6).

The results from the transplanted graft study showed that the issues of fibrosis and stenosis improved dramatically in the experimental group. Consequently, degassing treatment appeared to enhance the incorporation of the SIS mesh into the host tissue.

## 4. Discussion

In this study, we demonstrated that degassing SIS promotes cell sheet attachment in vitro. In a rabbit trachea patch repair model, the trachea defect repaired with the degassed SIS patch showed enhanced healing and reductions in fibrosis and luminal stenosis compared to the nondegassed control group. Our current study demonstrated the importance and benefits of degassing SIS, which has not been addressed in the literature previously.

SIS has been applied for more than two decades, as Clark et al. reported the use of intestine submucosa to repair the abdominal walls of dogs [6]. A later study confirmed that these bioscaffold materials functioned well to repair large ventral abdominal wall defects, and there was no evidence of local infection or other local detrimental pathology to any of the graft materials at any time point [23]. The short-term and long-term results from human studies were also satisfactory, even when used in contaminated or potentially contaminated surgical fields [24]. However, studies have also demonstrated that in some cases, especially in critically ill patients, the SIS mesh must be removed due to infection or reoperation [25]. In Clark’s study, the SIS bioscaffold showed more polymorphonuclear leukocytes in the SIS group at the 1-week time point than those in the other, non-SIS scaffold material groups, which raises concern for more significant foreign body reactions [6]. SIS has also been used for tracheal reconstruction in some studies. Gubbels et al. showed that SIS could be completely mucosalized, integrate into the surrounding tissues, produce minimal granulation, and support cartilage neoplasia using a vascularized perichondrial flap [26]. Bergonse et al. self-treated the submucosa of the small intestines of pigs for SIS implantation into rabbit tracheal defects with dimensions of 6mm × 8 mm (48 mm^2^) [27]. As described by the authors, after treatment, the acellular SIS was composed of collagen, elastin, glycoproteins, glycosaminoglycans, proteoglycans, and matricellular proteins, which is similar to the composition of the SIS graft we used in this experiment. The authors indicated that SIS facilitated neovascularization, epithelial remodeling, and immature chondrogenesis. However, the SIS alone could not ameliorate tracheal stenosis [27]. A promising way to increase the biocompatibility of SIS for various applications is to incorporate stem cells. Du et al. (2012) used monolayered mesenchymal stem cells (MSCs) combined with SIS to maintain airway patency, and the results were promising [28]. Nevertheless, the isolating and cultivating MSCs from adipose tissue to obtain the correctly differentiated cell has not always been sustainable and has not produced the desired results due to the decreased telomerase activity at higher cell passages. Even more importantly, long-term culture might lead to an increase in the probability of malignant transformation [29]. Alternatively, SIS can be modified to enhance cell attachment, and with increased cell attachment and migration, better healing and fibrosis reduction effects can be expected. Additionally, the SIS might be preattached to a respiratory epithelium cell sheet layer from the airway that can be easily harvested and cultured, such as a patient’s nose. Our previous study demonstrated the feasibility of fabricating an intact and transplantable cell sheet cultured from autologous rabbit nasal epithelial cells [21]. These nasal epithelial cell sheets appear to be functional and fully transplantable, which might serve as an ideal component in the abovementioned SIS scaffold applications to limit stenosis and preserve tracheal patency after transplantation.

To achieve the improved outcome mentioned above, the cytocompatibility of the SIS materials must be enhanced. Coating the surface with biocompatible substances such as collagen or hyaluronic acid is a commonly used protocol [30]. Surface modification with plasma can also be utilized to show significant improvement [31,32,33]. Nevertheless, none of these methods practically solves the problem that all of the current clinically available SISs are supplied in a dried form for storage at room temperature for a reasonable period of time. Inevitably, the prepared SIS is composed of dry ECM fibers with interlaced small air pockets that are initially filled with tissue fluid before being manufactured. Limited studies have addressed the impact of these SIS air pockets on wound healing. The degassing process, which is frequently used to eliminate microbubbles in meshes for many applications, is seldom addressed [34]. In a study by McKenna et al. on the fabrication of a dermal tissue engineering scaffold, degassing the scaffold (PLGA + E.C. solution) was found to be essential and the degassing process produced a morphology that was more consistent, increasing the suitability of the scaffold to support the growth of keratinocytes as well as promote skin tissue regeneration [35]. In contrast, in their study, the degassing process was emphasized to take place during the mesh manufacturing phase; we focused on applying the degassing process after manufacture. Using degassing protocols in a postproduction phase would allow physicians to further enhance the treatment effectiveness of a stock commercial product, which is essentially more clinically favorable. Whether degassing is performed during the pre- or postproduction phase, these studies demonstrated the importance of degassing and removing the dead space in the bioscaffold to increase the bioavailability of the material.

In our in vitro shaking/rinsing test, we observed better adhesion of the cell sheet on the SIS surface in the treated group. It should be noted that in our study, instead of using a direct cell seeding model, a cell sheet detachment and reattachment model was used to reveal the effectiveness of degassing the SIS surface. With direct seeding, the viability of the cells on the SIS varies significantly according to different cell types (unpublished data). We realized that under these conditions, we would actually be testing the survivability of the individual cells seeded on the SIS surface instead of testing the ability of the SIS surface to attach to the tissue. Therefore, the in vitro cell sheet reattachment model was chosen to more closely mimic physiological conditions, as SIS is typically placed in contact with living tissues in the clinic.

The benefits of degassing are also demonstrated in our in vivo study. Unlike in our previous study, where a nasal epithelial cell sheet was used as the scaffold lining in a tracheal patch defect model, in this study, pure SIS was used to repair the defect without any epithelial lining [21]. This allowed us to directly evaluate the effect of SIS degassing on tracheal defect reconstruction. Without the cell coverage provided by the inner lining, the tracheal wall defect was expected to undergo a primary healing process, in which the ability of the cells to attach and migrate would be directly reflected by the degree of healing, stenosis, and fibrosis. As expected, the animal defects repaired with the degassed SIS showed decreased degrees of stenosis and fibrosis at the healing site, implying that the degassing process effectively increased the primary healing ability. Nevertheless, the extent of fibrosis remained significant. Although the experimental animals will not experience mortality in this trachea wall patch defect model, if a segmental replacement or even transplantation is desired, the extent of stenosis/fibrosis reduced by the degassing process might not be sufficient to produce a favorable clinical outcome. Thus, utilizing the epithelial lining might be necessary for segmental replacements to prevent fatal stenosis [36]. Under these circumstances, efficient attachment of the cell sheet lining to the reconstruction scaffold will be necessary. As it has already been made commercially available and approved for use in humans, SIS might be one of the most readily available scaffolds in clinical practice. If the SIS can be preattached to the epithelial lining sheet, this hybrid scaffold–cell sheet might serve as an ideal transplant material for tissue repair, as SIS delivers mechanical strength for handling during surgery and the functional epithelial cell sheet lining provides functional coverage of the defect surface.

To achieve this notion, protocols intended to minimize the time needed for cell sheet adhesion as well as maximize the ratio of cell sheet adhesion onto the surface of the SIS scaffold are necessary. Using continuous negative pressure to remove the gas inside the scaffold and pulling the culture medium to fill the tiny pores on the surface of the SIS material entirely may help each part of the cell sheet have optimal exposure to nutrients, and the cell sheet may attach more quickly and firmly to the scaffold.

While the use of tissue-engineered hybrid “scaffolded” cell sheets might still take some time to be achieved, the effect of degassing revealed in this study can actually be used in clinical practice at the current stage. As degassing can be performed easily through negative pressure treatment, it is not difficult to perform in the operating room by simply applying the surgical suction system to an air-sealed chamber.

**Figure 7:**
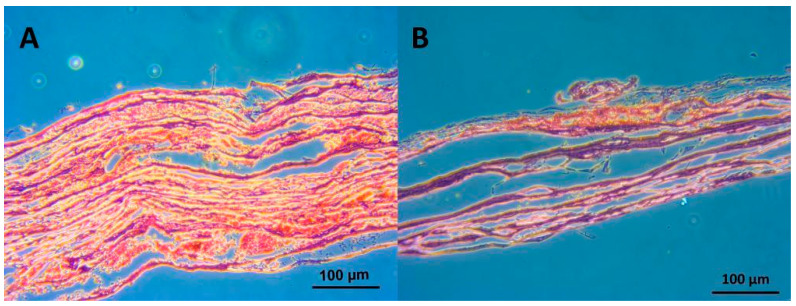
Demonstrations of SIS degassing with blood. (**A**) Microscopically, the red blood cells become interlaced with the SIS interfiber spaces after degassing. (**B**) When blood was poured on the SIS surface, the red blood cells aggregated on only the SIS surface and did not penetrate the SIS matrix after immersion for more than 30 min.

It is possible to optimize the surface of SIS materials by degassing and simply incorporating peripheral blood to enhance biocompatibility in vivo. In 2019, Sofu et al. used a chitosan-glycerol phosphate/blood implant (BST-CarGel^®^) mixed with peripheral blood that resulted in clinical and radiographic outcomes similar to those of a hyaluronic-acid-based cell-free scaffold for the treatment of focal osteochondral lesions of the knee joint [37]. Here, we recommend that physicians use a simple protocol by applying surgical suction in connection with a sterilized cup. The SIS can be placed in a sterilized dish and mixed with the blood gathered during the surgical procedure, and then the dish can be placed in a sterilized bag or chamber and connected to the surgical suction system, which would easily degas the SIS and allow the peripheral blood to fill the air pockets within it. We observed dramatic SIS softening after 15–30 min of degassing treatment. Microscopically, the red blood cells were observed to be interlaced with the SIS interfiber spaces after degassing (Figure 7A). Then, when the blood was poured on the SIS surface, the red blood cells aggregated on only the SIS surface and did not penetrate the SIS matrix even after immersion for more than 30 min (Figure 7B).

This study has several limitations. First, a patch tracheal defect repair model was used, and a relatively small defect that was below the fatal threshold was created and repaired. Thus, this study did not fully mimic the clinical conditions of tracheal implantation. Although degassing might have some positive impacts on partial tracheal repair, degassing would be of less clinical value if this protocol cannot be applied in circumferential segmental repair or transplantation. Our next step will be to test the degassing protocol in a whole tracheal segmental transplantation model to evaluate the true effectiveness of this degassing procedure. Additional evaluations of respiratory dynamics should also be considered. Second, while the removal of the air pockets in the SIS seems to be the critical feature of this study, it is difficult to observe cell-to-surface contact behavior consecutively in real time, as the SIS is a nontransparent material with a specific thickness, making it difficult to observe by light microscopy. A noncytotoxic alternative to scanning electron microscopy must be used to more clearly demonstrate that the air pockets become obstacles to the living cells when the cells are trying to attach, migrate, and proliferate.

Third, the pressure and pretreatment time needed to remove a substantial number of air bubbles inside the SIS material to facilitate the adhesion and proliferation of the respiratory epithelial cell sheets were not precisely determined. The degassing time of approximately 30 min in our current protocol seemed to be acceptable to maintain a functional cell sheet, but optimization is worth further investigation.

Last, the potential of developing a wound infection after degassing the SIS should always be kept in mind, because after removing the air bubbles from the materials, the bioavailable spaces created can be used by microorganisms. Thus, whether this degassing process causes a higher tendency to develop subsequent infection needs to be further explored, mainly since SIS is intended to be used in contaminated surgical fields. In our study, we have observed no signs of inflammation or infection under H&E staining. However, an additional check of inflammatory markers such as the cytokines might help identify the concerns mentioned above.

Despite these limitations, we believe that the degassing process, which helps to remove air bubbles from inside a porous material, plays a pivotal role in increasing cell-material adhesion and biocompatibility and thus might be a vital component for the clinical applications of SIS. SIS was approved by the FDA a long time ago, which makes it easy to purchase and to apply in humans. Most importantly, the degassing process can be easily performed in almost all operation rooms as long as a suction device is available. Surgeons might consider immersing the SIS in clean body fluid or saline under negative pressure for a short period of time before being applied to the desired surgical field. The increased biocompatibility gained by using this simple treatment might significantly enhance the effectiveness of SIS without the need for complicated, time-consuming modifications.

## 5. Conclusions

In conclusion, degassing the SIS mesh significantly promoted cell sheet attachment in vitro. In the tracheal patch repair model, degassed SIS significantly promoted wound healing by reducing luminal fibrosis and stenosis compared to the nondegassed control mesh. These results suggest that degassing the SIS might be a simple and effective way to improve its biocompatibility.

## 6. Patents

Tseng How has patent #US9,546,349 B2 licensed to Taipei Medical University.

## Data Availability

Not applicable.
